# Leprosy in Spain: A Descriptive Study of Admissions at Fontilles Sanatorium between 1909 and 2020

**DOI:** 10.3390/tropicalmed9060130

**Published:** 2024-06-11

**Authors:** Cristina Juan, Lourdes Lledó, Miguel Torralba, José Ramón Gómez, Consuelo Giménez

**Affiliations:** 1 Department of Biomedicine and Biotechnology, Faculty of Pharmacy, Alcalá University, 28805 Alcalá de Henares, Madrid, Spain; lourdes.lledo@uah.es (L.L.); consuelo.gimenez@uah.es (C.G.); 2 Internal Medicine Unit, Department of Medicine and Medical specialities, IDISCAM, Faculty of Medicine, Alcalá University, 28805 Alcalá de Henares, Madrid, Spain; miguel.torralba@uah.es; 3 Sanatorium San Francisco de Borja, Fontilles, 03791 Alicante, Spain; jrgomez@fontilles.org

**Keywords:** leprosy, *Mycobacterium leprae*, Spain, time trend

## Abstract

Background: The study aimed to characterize patients with leprosy admitted to Fontilles throughout the 20th and 21st centuries, focusing on differences across three periods (I, II, and III). It also explored variables linked to patient survival. Methods: This was a retrospective descriptive study analyzing the medical records of Fontilles patients from 1909 to 2020. It assessed 26 clinical, sociodemographic, and temporal variables (*n* = 2652). Results: Most patients were male, single, multibacillary (MB), and farmers, from Andalusia and the Valencian Community. The origin of patients shifted over time towards being mostly foreign-born in period III. More than a half were previously admitted and had family members with leprosy. While leprosy reactions decreased over time, neurological symptoms were increasingly diagnosed. The age at onset, admission, and death increased progressively over time. The survival of patients with leprosy at Fontilles depended on the age at admission and the period. Conclusions: Improved knowledge, services, and awareness regarding leprosy led to increased age at onset and more favorable outcomes. The prolonged time between symptom onset and diagnosis indicates that leprosy is still a neglected disease. Although MB forms are more severe, leprosy classification did not significantly impact the survival rates of patients at Fontilles.

## 1. Introduction

Leprosy is a chronic infectious disease caused by *Mycobacterium leprae* and *lepromatosis*, an acid-fast bacillus characterized by its prolonged incubation period. It primarily affects the skin, the peripheral nervous system, and the mucosa of the upper respiratory and digestive tract. The transmission of *M. leprae* occurs by inhalation, directly and/or indirectly, but infection may also occur through the skin. Although the bacillus usually originates from multibacillary patients, it may also come from the environment and animals. Reservoirs include armadillos, soil, and water sources [1,2,3,4,5,6,7,8,9]. 

Concerning the epidemiology of leprosy, approximately 200,000 cases are diagnosed each year in almost 120 endemic countries. Most cases originate from Indonesia, Brazil, and India [2,4,7,10,11,12,13]. Given the significant impact of Neglected Tropical Diseases (NTDs), including leprosy, the World Health Organization (WHO) launched a roadmap in 2020 aiming to advocate, among other priorities, the acquisition of knowledge about NTDs by fostering cross-sectoral collaboration and addressing stigma through a human rights lens [4,14,15,16]. 

Throughout history, people with leprosy have been stigmatized and usually lived isolated in colonies, as in Spain during the 20th century [17,18,19,20]. The Sanatorium of Fontilles—also knowns as San Francisco de Borja—was a leprosy hospital opened in Alicante (Spain) in 1909, which became a model colony for patients with leprosy [21,22,23,24]. The sanatorium allowed the isolation of patients when leprosy was a public health problem in Spain, probably due to the poor socioeconomic and health conditions in this area. In 1943, there were four regions in Spain with a high incidence of leprosy: Levante (including Catalonia, Valencia, and Murcia); Andalusia; Galicia; and the Canary Islands [13,25]. The sanatorium housed approximately 3000 individuals, hosting patients from all the above regions due to the lack of infrastructures to isolate patients [23,24]. 

**Figure 1:**
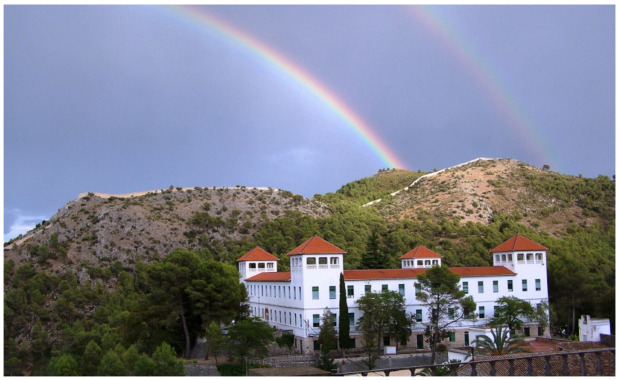
Sanatorium of Fontilles in 2020.

This paper aims to deepen the understanding of leprosy. Firstly, it describes the demographic and clinical characteristics of patients admitted at Fontilles (see Figure 1). Secondly, it compares mortality risks across three distinct periods to identify any significant differences.

## 2. Materials and Methods

### 2.1. Design

The study is a retrospective descriptive analysis of the medical records of 2652 patients admitted to Fontilles between 1909 and 2020. These records are housed within the historical archive of the institution.

### 2.2. Data Collection and Data Grouping 

From 2009 to 2011, three workers spent three hours per week gathering temporal, sociodemographic, and clinical data. Afterwards, further data on the background of patients were collected. A final examination of the data was undertaken in November 2023. Anomalous figures were addressed, terminology grouped, and new variables introduced. Although the International Classification of Diseases has significantly improved over time, the classification of the causes of death over the studied periods remained difficult, given that numerous different terms were used to refer to potentially the same cause of death. 

### 2.3. Statistical Analysis

The data were initially imported into a database in Excel-2019 (Microsoft^®^) and analyzed in SPPS version 27. For the descriptive analysis, frequencies, medians, and ranges were used, depending on the nature of the variables. The Chi-squared test was used to compare categorical variables (or the Fisher exact test, if necessary). A *p*-value of less than 0.05 was judged statistically significant. Student’s t-test was used to compare two groups, while analysis of variance (ANOVA) was used for comparisons involving more than two groups. For risk estimation, the odds ratio (OR) was calculated with a 95% confidence interval (CI). 

Since all the patients studied had leprosy, and considering that this disease was not their primary cause of death, this study also aims to compare their causes of death to those of the general Spanish population in the 20th and 21st centuries. It draws upon the research on Spanish mortality in the 20th century conducted by the National Institute of Health Carlos III (ISCIII) [26]. The survival analysis of patients was calculated by recording mortality patients who were at least admitted to Fontilles, regardless of whether they died inside or outside the sanatorium. Given that leprosy is not known to be a fatal disease, this study aimed to establish whether other risk factors, such as leprosy classification, age at admission, and country of origin, were associated with the death of patients with leprosy. To achieve this, a multivariate analysis was performed using Cox regression models with dependent variables that were time-dependent and hazard ratios (HR), with a 95% CI. 

### 2.4. Ethical Considerations

Initially, the database was provided with a Fontilles-specific identification number, but it did not include any personal data. The study protocol received approval from the Institutional Review Board for Human Research (IRB) at the University Hospital of Guadalajara (Spain) on 11 July 2023, with MINUTES NUMBER 7/2023. During the treatment and analysis of the data, ID numbers were removed and patients were identified with random numbers ranging from 1 to 2652.

### 2.5. Studied Variables

#### 2.5.1. Temporal

To facilitate the examination of Fontilles attendance, the timeframe was divided into three distinct periods. Period I runs from 1909, the year the sanatorium opened, to 1945, when sulfone (the first antibiotic for leprosy treatment) arrived at Fontilles. Period II spans the period 1946 to 1982, when the WHO introduced multidrug therapy (MDT). Finally, period III runs from 1983 to 2020.

#### 2.5.2. Sociodemographic

The sociodemographic variables were gender, civil status, most common occupation (based on the National Classification of Economic Activities, 2009), age at admission, paper medical records, community of origin, country of origin, previous history of migration, previous admission to other facilities, family members with leprosy, neighbors or acquaintances with leprosy, number of children per family, and number of children per household without leprosy.

#### 2.5.3. Clinical

The clinical variables were classification, mortality (alive/deceased), age at death, age at onset of leprosy, time interval between symptom onset and diagnosis (years), active surveillance, duration of active surveillance (years), previously treated patients, treatments, first symptoms at onset, typology of skin symptoms, location of skin symptoms, and cause of death. 

The clinical classification of leprosy evolved over time. In 1916, dermatologist Cazenave made a distinction between black and white leprosy. In 1953, during the Congress of Leprosy in Madrid, a classification system was designed to differentiate between malignant forms (lepromatous, LL) and benign forms (tuberculoid, TT), adding the intermediate (I) and mid-borderline (BB) categories. In 1968, Ridley and Jopling introduced borderline tuberculoid (BT) and borderline lepromatous (BL) forms, eliminating indeterminate leprosy. In 1981, the WHO classified patients with fewer than five skin lesions as paucibacillary (PB) and those with more than five as multibacillary (MB). In 2007, this classification was improved: PB cases were redefined to include people with 1 to 5 skin lesions without the presence of bacilli in skin smears or biopsies, while MB cases referred to people with more than 5 skin lesions, or with nerve involvement, or with the presence of bacilli in a slit-skin smear, irrespective of the number of skin lesions [1,2,4]. The leprosy classification adopted in this study was that defined by the WHO in 1981. This means that, previously, LL, BB, BT, and BL were considered MB, and the only PB form considered was TT. 

## 3. Results

The study encompassed a cohort of 2652 individuals spanning 111 years, with 1774 paper medical records available, representing 66.9% of all the patients admitted to the sanatorium. The rest of the data were obtained from the registration books of Fontilles. The median age at admission was 37.5 years (IQR = 27.5–52.5); 63.1% were male and 36.9% were female. MB forms predominated over PB forms; 89.9% and 10.2%, respectively. The median age of survival in period I was 5.4 years (IQR = 2.1–13.6). In period II, it increased to 28.2 years (IQR = 11.5–45.2), and in period III, it decreased to 17.5 years (IQR = 9.1–19.1). In addition, the median age at death was 55.3 years (IQR = 38.7–70.4).

**Figure 2:**
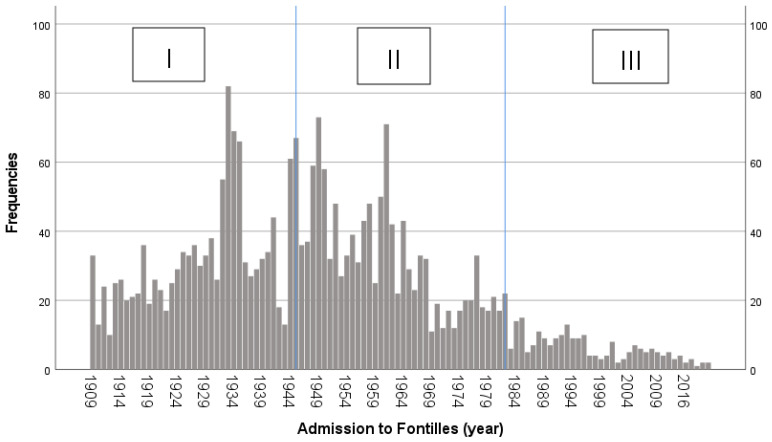
Admissions of patients with leprosy to Fontilles over time.

In period I, 1228 (46.5%) patients were admitted; in period II, 1188 (45.0%); and in period III, 223 (8.5%). The distribution of admissions is shown in Figure 2.

Most people were single (54.4%), mainly from the Valencian Community (41.7%), Andalusia (37.4%), and Catalonia (5.0%). Only 2.8% were foreign-born. Additionally, a total of 8.3% reported a previous history of migration. The National Classification of Economic Activities 2009 comprises a total of 21 professional groups, indicated as A to U and involving all types of existing jobs. The most common categories were A (agriculture, livestock, forestry, and fishing) and T (activities of households as employers of domestic personnel; activities of households as producers of goods and services for own use), with a 40.1% and 26.6%, respectively. 

From the total number of patients, 59.8% had been admitted to other facilities, usually a hospital, before arriving to Fontilles. Regarding family members with leprosy, 58.0% had at least one person in their household, usually the mother, while 35.5% had neighbors or acquaintances with leprosy. The median number of children per household was three (IQR = 0–3) and, in most cases, two of them (IQR = 0–5) did not have leprosy. The median age at onset of leprosy was 26.5 years (IQR = 18.5–40.5) and the median time interval between symptom onset and diagnosis was 6 years (IQR = 6–13). After admission, patients were monitored by Fontilles for an average of 4.9 years (IQR = 1.3–14.0). It is worth noting that 76.6% of the patients had been treated before arriving to Fontilles. The most common treatment during period I was antileprol (commonly known as chaulmoogra oil), administered to 11.8% (145/1228) of patients. In period II, patients were commonly treated with DDS (dapsone), which represented 15.8% (118/1188) of cases. In period III, most patients were treated with MDT therapy. In addition, 36.1% of patients were under active surveillance at clinics or hospitals in Spain. 

The initial symptoms at onset were predominantly skin-related (31.0%), followed by neurological symptoms (16.0%) and leprosy reactions (9.7%). Regarding the typologies of skin symptoms, nodules (also known as lepromas) were the most common (51.0%), followed by macules (35.7%) and alopecia (6.4%). Skin symptoms were predominantly found on the limbs in 95.0% of the cases. 

The cause of death of patients at Fontilles could be confirmed with a certain degree of certainty in 31.2% of cases. However, it remained unclear whether patients died during their stay at Fontilles or after their discharge. From the available data, the most prevalent causes of death included cardiovascular diseases (22.3%), kidney diseases (19.7%), certain leprosy complications such as cachexia, enteritis, or leprosy reactions (18.6%), other diseases (13.0%), liver or bowel diseases (9.5%), respiratory diseases (9.1%), and tumors (7.7%). Other diseases comprised 170 different diseases, which individually did not reach 25 cases. 

### 3.1. Differences between Periods

An association was made between periods and other qualitative variables (see Table 1). The *p*-value refers to a statistically significant association between the mentioned variables.

Period I had a higher number of patients from the Valencian Community, while period II and period III saw the most patients from Andalusia (*p* < 0.001). In addition, the percentage of foreign-born patients increased significantly over time (*p* < 0.001). Category A had a higher percentage of patients in period I, but decreased over time, while category T increased over time (*p* < 0.001). The percentage of patients previously admitted to other facilities before arriving at Fontilles increased over the periods, reaching 100% in period III (*p* < 0.001). The prevalence of family members with leprosy increased over time (*p* = 0.012), while the prevalence of neighbors or acquaintances with leprosy progressively declined (*p* < 0.001). A considerable percentage of patients had been treated before arriving at Fontilles, reaching 100% in period III (*p* < 0.001). 

The onset of the disease with skin symptoms was the most prevalent throughout all periods, especially in period II. Leprosy reactions were most common in periods II and III, followed by neurological symptoms in period III (*p* < 0.001). Additionally, nodules or lepromas were more prevalent in period III, while macules, other skin symptoms, alopecia, unknown symptoms, and several symptoms (more than one category) reduced over time (*p* < 0.001). All categories related to the location of symptoms, except for the limbs, increased in number as time elapsed (*p* < 0.001). Period I had a higher number of deaths compared to the subsequent periods and, therefore, most surviving patients were grouped together in period III (*p* < 0.001). Certain leprosy complications (cachexia, enteritis, or leprosy reactions), kidney diseases, respiratory diseases, liver or bowel diseases and cardiovascular diseases steadily decreased over time, but tumors and other diseases increased in prevalence in period III (*p* < 0.001).

The association between periods and quantitative variables are shown in Table 2. The *p*-value indicates that the associations between the variables cannot be justified by chance and therefore the null hypothesis can be rejected. 

The age at admission significantly increased over time (*p* < 0.001). The number of children per family increased from period I to period II, before stabilizing in period III (*p* < 0.001). The number of children without leprosy per household increased from period I to period II, but decreased in period III (*p* < 0.001). The age at onset of leprosy started increasing over time (*p* < 0.001). The time interval between symptom onset and diagnosis markedly increased from period I to period III, but decreased in period II (*p* < 0.001). The duration of active surveillance increased in period II, compared to period I, while in period III it considerably decreased (*p* < 0.001). In addition, the age at death increased over time (*p* < 0.001).

### 3.2. Survival of Patients

With the aim of understanding the variables associated with patient survival at Fontilles, a Cox regression model was conducted with mortality as the dependent variable and country of origin (Spain vs. other), gender, leprosy classification, age at admission (for every decade), and period as independent variables. The findings are summarized in Table 3.

Patients in period II showed a 49% decrease in mortality compared to period I. Similarly, patients in period III showed a 57% decrease in mortality. On the other hand, for every decade in age at admission, the mortality rate increased by 64%. Such conclusions were independent of gender, country of origin (Spain vs. other), and leprosy classification. 

## 4. Discussion

The overall findings of the study revealed that the majority of patients were middle- aged single males with MB leprosy. Most admissions occurred during periods I and II and the diagnosis often occurred late after symptom onset. Regarding the first symptoms at onset, leprosy reactions were infrequent in period I, while neurological symptoms were more prevalent in period III. The average duration of active surveillance of patients was significant, and both the age at admission and age at death increased over time. Patients died from the same causes observed in Spain in general during the 20th and 21st centuries. The progression of periods and the age at admission significantly impacted patient survival at Fontilles, irrespective of gender, country of origin, and leprosy classification.

The male predominance is also mentioned by other authors [25,27,28,29,30,31,32,33,34,35]. This male predominance could be related to the under-diagnosis of women: their physical examination was less thorough due to cultural customs and limited access to health services [2,31]. However, studies in Africa suggest that women there are more affected by leprosy than men [31,36,37]. A higher prevalence of MB patients is also observed by others, even those who believed in an under-diagnosis of MB cases due to a lack of experience among clinicians [37]. Contradictorily, several publications report an under-diagnosis of PB forms, which is unlikely to be true [38]. A high percentage of patients with leprosy were single, which is consistent with other studies [25,39]. In addition, agriculture was typically found to be the primary occupation in several studies, which is associated with a lower socioeconomic status and educational level, resulting in limited access to health care [25,27,40,41]. The evolution of the occupation categories was linked to more access to education, improvements in qualifications, and the incorporation of women into the labor market. Additionally, the percentage of patients who had a previous history of migration is similar to prior studies [25].

The average age at onset of leprosy is consistent with other studies, considering the incubation period of leprosy between 5 and 10 years [2,25,27,28,29,30,31,34,35,37,41]. The age at onset was similar to other studies and covers a period characterized by an intense social and work life, increasing people´s exposure to the disease [25,30,32,34,36,37]. There were few cases of pediatric patients throughout the studied periods, suggesting a decrease in the transmission of *M. leprae* over the three periods [2,34,37]. The significant time interval between symptom onset and diagnosis indicates a late diagnosis. This is probably caused by the non-specific symptoms of leprosy and the lack of disease control measures among the population at risk. Importantly, period III registered the longest time interval between symptom onset and diagnosis. This is likely due to leprosy being an NTD, and the delay in diagnosis contributes to the risk of developing disabilities [7,32,42]. Another reason could be that this last period presented a higher number of foreign-born patients who experienced more difficulties accessing healthcare, which resulted in a delayed diagnosis [7,9,10,43,44,45].

The vast majority of admissions occurred during periods I and II, which is congruent with the findings of Urbina et al. (1997) who conducted a study at the Sanatorium of Trillo (Spain). The decline in patients in period III is consistent with the global decrease in cases due to economic growth and development [8,35,36,37,40,43,44,45]. Moreover, the incidence rates steadily declined in Spain after introducing consultation with mobile clinics around 1950, facilitating the on-site treatment of patients living in remote villages, and the introduction of MDT in 1980 [13,37,40,42,46]. Regarding communities of origin, Urbina et al. (1997) gathered comparable data. Andalusia held the first place due to the highest rate of leprosy patients from 1950 to 2000, in a time of significant infrastructure scarcity. The Valencian Community ranked second due to its proximity to Fontilles, as mentioned by other authors [40,46,47,48]. In addition, Catalonia occupied the third place, taking into account the migratory movements from Extremadura, which reported a large number of leprosy cases. Furthermore, prior to 1982, leprosy patients diagnosed in Catalonia were frequently referred to Fontilles. Analyzing the communities of origin over the periods, at the beginning, patients came from the Valencian Community because Fontilles was created in response to a local public health emergency [48]. The higher incidence of leprosy in Andalusia explains why most patients came from this region later on [40]. Furthermore, up until 1950, a hospital in Granada was the only one available, although the capacity was limited [46,47,49]. Migratory patterns explain that foreign-born patients were mainly recorded throughout period III [7,9,10,44,45].

The fact that more than a half of patients were admitted to other facilities before arriving to Fontilles may reflect the challenge of being diagnosed [39]. Additionally, over the periods, healthcare facilities became more accessible, increasing the probability of being admitted to other facilities before arriving at Fontilles. In other studies, the percentage of family members with leprosy is lower than in this study, around 30% [14]. It should be underlined that the percentage of family members with leprosy increased over the periods, probably due to the discovery that leprosy was not inherited and the increase in community awareness. The stigma towards leprosy gradually diminished and people did not feel the need to hide their leprosy status. In addition, the transmission of *M. leprae* depends on individual immune responses and environmental conditions [9,34]. The decline in the number of neighbors or acquaintances with leprosy is explained by a lower incidence of leprosy and the decrease of close contact. The current study found that the average number of children per household was two, assuming these are nuclear families, which is closely aligned with the findings of a study in Jaen [49]. The pattern of the evolution of children per household corresponds to the high natality registered at the end of the Spanish Civil War. Based on birth rates, the proportion of children without leprosy across periods decreased over time, possibly because the spread of leprosy depends on the individual genetic predisposition.

A higher proportion of patients, especially in period I, were treated prior to arriving at Fontilles, which was associated with unsuccessful treatment. In addition, most patients in period III were treated with MDT as recommended by the WHO. The absence of leprosy reactions in period I can be linked to the lack of a successful treatment while a higher percentage of neurological symptoms in period III was linked to better detection methods. Improvements in treatment explain the decrease in most symptoms over the periods, while a greater awareness and responsibility for health among individuals is linked to other location of symptoms. Similar studies found that skin symptoms on the limbs are the most common [27,38,50].

The low percentage of patients found through active surveillance was due to stigma, keeping patients away from health authorities. In addition, the long period of active surveillance after diagnosis is linked to leprosy reactions, which could continue even after treatment was completed [2]. Patients in the latter period developed leprosy at an older age due to a reduction in disease transmission [8]. Moreover, the increase in the age at admission over time is similar to other findings after the emergence of effective treatments and the delayed diagnosis of leprosy as a neglected tropical disease [4,32,43]. The increase in the age at death over time, as shown in similar studies, can be related to the general socioeconomic development as well as the advent of fixed-dose combination therapy [25].

The lower median age of survival in period II compared to period III can be attributed to the older age and more advanced stages of leprosy among patients admitted during period III. Describing the available data on the causes of death among patients with leprosy at Fontilles, the decrease in kidney, genitourinary, respiratory, and liver or bowel diseases in period I was consistent with the mortality causes in Spain throughout the 20th century. In addition, cardiological diseases showed an increase in prevalence during period III, less pronounced than expected, but it remained the leading cause of death overall [26,51]. Certain leprosy complications (cachexia, enteritis, and leprosy reactions) showed a reduction, whereas tumors and other diseases increased over the periods given the medical developments during the 20th century. In addition, a more accurate classification of diseases was implemented. In terms of case fatality among patients at Fontilles, advances in therapies, changes in public policies, and fluctuations in leprosy prevalence influenced the link between period progression and patient survival, reducing patient mortality. Furthermore, age at admission is another factor influencing survival. This is regulated by a variety of biological reactions and treatment success variation among stages of life.

This study encountered typical limitations of historical research, such as complex data collection spanning a long period, incomplete data records, challenges in classifying leprosy into MB and PB forms, and the lack of data on rifampicin and MDT, which hindered mortality assessment. Furthermore, pertaining to the causes of death, laryngeal obstruction, which was common during the early study, did not appear in the medical records. There is no information available on autopsies to determine the real causes of patients’ death. In addition, the loss of paper medical records linked to the beginning of the Second Republic in Spain (1931–1939), the Spanish Civil War (1936–1939), some administrative changes, and a fire that apparently broke out, contributed to the loss of data [24]. On the other hand, some of the major strengths of this study were the extensive number of medical records analyzed, the long follow-up period considered, and the study of this neglected disease over 111 years. It was possible to analyze the factors independently associated with mortality using multivariate techniques.

## 5. Conclusions

The pattern of patients admitted to Fontilles corresponds with the prevalence of leprosy in Spain throughout the 20th and 21st centuries. Significant improvements in knowledge, services, and awareness about leprosy over time contributed to the increase of the age at onset, and favorable admission and death statistics. Spain is currently a non-endemic area for leprosy, and thus, in period III, most patients were foreign-born. Leprosy remains a neglected disease, as evidenced by the prolonged time interval between symptom onset and admission. Although the severity of MB forms is higher than that of PB forms, leprosy classification did not have an impact on the death of patients with leprosy at Fontilles.

## Figures and Tables

**Table 1:** Association between qualitative variables and each period of the study.

		Period						
Characteristics/Variables	Category	I		II		III		*p*-Value *
		N	%	N	%	N	%	
Community of origin	Andalusia	258	21.1	606	51.8	106	51.7	<0.001
	Castilla la Mancha	32	2.6	33	2.8	3	1.5	
	Catalonia	53	4.3	77	6.6	1	0.5	
	Valencian Community	766	62.6	288	24.6	31	15.1	
	Extremadura	17	1.4	38	3.2	5	2.4	
	Region of Murcia	46	3.8	55	4.7	9	4.4	
	Foreign countries	4	0.3	17	1.5	32	15.6	
	Others	48	3.9	56	4.8	18	8.8	
Country of origin	Other	5	0.4	23	1.9	45	20.2	<0.001
	Spain	1223	99.6	1165	98.1	178	79.8	
Most common occupation (National Classification of Economic Activities, 2009)	T (households as employers of domestic personnel)	137	28.1	345	46.5	38	49.4	<0.001
	A (agriculture, livestock, forestry, and fishing)	350	71.9	397	53.5	39	50.6	
Previous admission to other facilities	Yes	175	52.6	371	59.7	58	100.0	<0.001
Family members with leprosy	Yes	207	59.3	513	55.5	116	67.4	0.012
Neighbors or acquaintances with leprosy	Yes	168	48.1	300	32.5	47	27.3	<0.001
Treatment	Yes	216	64.1	496	79.7	99	99.0	<0.001
Active surveillance	Yes	116	38.0	137	34.5	1	100.0	0.222
First symptoms at onset	No category	848	69.1	70	5.9	18	8.1	<0.001
	Leprosy reactions	68	5.5	163	13.7	25	11.2	
	Edema	12	1.0	43	3.6	1	0.4	
	Ear, nose, and throat symptoms	9	0.7	31	2.6	3	1.3	
	Neurological symptoms	74	6.1	288	24.2	100	26.9	
	Skin symptoms	184	15.0	536	45.1	16	44.8	
	Several (more than one category)	32	2.6	57	4.8	16	7.2	
Typology of skin symptoms	Nodules or lepromas	59	27.7	294	50.7	110	95.7	<0.001
	Macules	101	47.4	222	38.3	1	0.9	
	Other skin symptoms	10	4.7	4	0.7	3	2.6	
	Alopecia	13	6.1	45	7.8	0	0.0	
	Unknown symptoms	13	6.1	5	0.9	1	0.9	
	Several (> 1 category)	17	0.8	10	1.7	0	0.0	
Location of skin symptoms	Disseminated	4	1.9	0	0.0	5	4.3	<0.001
	Trunk, abdomen, buttocks, or back	1	0.5	1	0.2	4	3.5	
	Limbs	192	90.1	578	99.7	94	81.7	
	Unknown	6	2.8	1	0.2	8	7.0	
	Face, eyebrows, or auricular pavilion	10	4.7	0	0.0	4	3.5	
Mortality	Alive	173	18.6	711	63.8	167	84.8	<0.001
	Deceased	755	81.4	404	36.2	30	15.2	
Causes of death	Kidney diseases	75	17.6	86	22.9	2	8.0	<0.001
	Cardiovascular diseases	99	23.2	828	21.9	4	16.0	
	Leprosy complications	124	29.1	28	7.5	2	8.0	
	Liver or bowel diseases	30	7.0	49	13.1	0	0.0	
	Respiratory diseases	43	10.1	31	8.3	0	0.0	
	Tumors	14	3.3	45	12.0	5	20.0	
	Others	41	9.6	54	14.4	12	48.0	

* p-value calculated using Chi-square test; N: Number.

**Table 2:** Association between quantitative variables and each period of the study.

Characteristics/Variables			Period				*p*-Value *
	I		II		III		
	No	Mean (SD)	No	Mean (SD)	No	Mean (SD)	
Age at admission	1069	38.1 (15.6)	1181	40.4 (16.5)	218	51.0 (17.3)	<0.001
Number of children per household	1226	1.6 (2.8)	1183	5.4 (3.1)	216	4.13 (3.4)	<0.001
Number of children per household without leprosy	1226	1.2 (2.3)	1183	4.23 (3.1)	216	2.82 (3.3)	<0.001
Age at onset of leprosy	385	26.0 (13.5)	1156	31.7 (16.1)	211	32.7(17.7)	<0.001
Time interval between symptom onset and diagnosis (years)	385	10.3 (9.0)	1155	8.4 (9.2)	211	18.0 (18.0)	<0.001
Duration of active surveillance (years)	949	8.4 (11.5)	1146	12.9 (13.8)	196	3.8 (5.2)	<0.001
Age at death	728	49.2 (17.6)	306	62.6 (16.8)	30	73.0 (12.2)	<0.001

* sig bilateral value calculated using ANOVA.

**Table 3:** Survival analysis of patients at Fontilles.

Characteristics/Variables	B	*p*-Value	HR	95% CI HR
Country of origin (Spain vs. other)	0.266	0.519	1.305	0.581–2.932
Gender male vs. female	0.045	0.553	1.046	0.901–1.216
Leprosy classification (MB vs. PB)	0.180	0.215	1.197	0.901–1.59
Age at admission (for every decade)	0.497	<0.001	1.644	1.560–1.734
Period I (reference)	NA	<0.001	NA	NA
Period II (vs. period I)	−0.672	<0.001	0.511	0.438–0.595
Period III (vs. period I)	−0.831	<0.001	0.436	0.293–0.648

MB: multibacillary, PB: paucibacillary; B: Coefficient; HR: Hazard ratio; CI: Confidence interval; NA: not applicable; The reference for country of origin is ‘other’, the reference for gender is ‘female’, and the reference for leprosy classification is ‘PB’.

## Data Availability

All the data for this study will be available upon reasonable request.
